# Costs and savings associated with a pharmacists prescribing for minor ailments program in Saskatchewan

**DOI:** 10.1186/s12962-018-0159-y

**Published:** 2018-11-22

**Authors:** Ellen Rafferty, Mohsen Yaghoubi, Jeff Taylor, Marwa Farag

**Affiliations:** 1 School of Public Health, University of Saskatchewan, Saskatoon, Canada; 2 College of Pharmacy and Nutrition, University of Saskatchewan, Saskatoon, Canada

**Keywords:** Canada, Pharmacist, Pharmaceutical services, Physician, Nurse practitioners, General practice, Primary health care, Health policy, Conflict of interest, Economics

We thank the author of the letter for reading and drawing attention to our work. We think that our knowledge and understanding of any issue is advanced by actually producing research that stimulates discussion and debate, and the author of the letter proved that our research did just that. The author of the letter raises issues about: (1) State of the evidence on pharmacist prescribing programs’ effectiveness and safety; (2) Study design; (3) Valuation of inputs; (4) Findings; and (5) Policy Implications. We will respond to the points raised by the author of the letter sequentially in the following paragraphs.

## State of the evidence on pharmacist prescribing programs’ effectiveness and safety

We disagree with the author of the letter’s view on the safety, efficiency and effectiveness of pharmacist prescribing programs. The fact is most of the literature, including the work of Mansell et al. [1], which the author mentions, reports positive outcomes. Mansell et al. reported 81% symptom improvement for participants in Saskatchewan [1]. A systematic review by Paudyal et al. [2] found that the proportion of patients reporting complete resolution of symptoms after an index Pharmacy-based Minor Ailment Schemes consultation ranged from 68 to 94%.

Two of the papers to which the author of the letter referred [3, 4], did not specifically examine pharmacist prescribing for minor ailments programs (PPMA) and hence their findings are not directly relevant to our study. However, we do acknowledge that they speak to overall pharmacist competence, an aspect that is very important to the discussion of pharmacists taking on new duties.

The third study which the author of the letter referenced is a study from the UK examining a PPMA program; none of this study’s findings suggest that a physician visit would be an improvement over pharmacist prescribing since there is no comparator [5].

The purpose of our paper was not to evaluate the clinical outcomes of the program, but rather to evaluate the costs and savings associated with the PPMA, While, we agree with the author of the letter that more research assessing the outcomes and quality of pharmacist prescribing programs is critically needed, we think that our role as researchers is to use the best available evidence and not wait for the perfect conditions to conduct research. Otherwise, little research may actually be produced. To our knowledge, our study is the only study specifically examining costs and savings of such programs in Canada, published in a peer-reviewed journal.

There are four main journal articles and one working paper from the UK specifically examining costs and outcomes of pharmacist prescribing programs. Watson et al. found positive health-related outcomes and substantially lower costs with pharmacy consultations for minor ailments [6]. Wagner et al. [7] found higher uptake of the minor ailment program in the most deprived areas, suggesting pharmacist prescribing programs may be particularly beneficial to those most at risk of health problems and those with limited access to health care. Bojke et al. [8] showed a reduction in the proportion of physician visits that were for minor ailments. Moreover, Baqir et al. [9] estimated the effect of PPMA on reducing health care cost and found that the PPMA program saved the local health authorities about £6739 per month. Finally, a comprehensive working paper from the Welsh Government Social Research estimated cost, effect and return on investment of implementing PPMA in UK and indicated that the estimate of the benefits of delivering Choose Pharmacy over a 5 year period ranges from £5 million to £75 million in UK [10]. If new research findings indicating negative impacts on quality and outcomes of these pharmacist prescribing programs become available in the future, then new research should be conducted taking this new evidence into account. However, in conclusion, our findings are consistent with other published peer-reviewed work in this area and the evidence is overwhelmingly positive in spite of the author of the letter’s view on the matter.

### Study design

Our study falls under the category of economic impact analysis. Economic Impact Analysis (EIA) is a methodology for evaluating the impacts of a project, program or policy (e.g. PPMA program) on the economy of a specified region (costs and savings to society, and to the health care budget) [11].

### Valuation of inputs

As for the input estimates, we can understand that the author of the letter might disagree with how we valued certain inputs, as this type of variation in estimates is inevitable. This is why we conducted extensive sensitivity analyses in our original study. Even though we provided detailed references and sources for each of the inputs we used in the paper, we discuss the specific inputs that the author of the letter highlighted, in detail, in the following paragraphs.

#### Cost of a physician visit

We obtained the cost of a physician visit from the Saskatchewan schedule of benefits as the cost of an exploratory GP appointment ($67.00) [12]. We are not sure why the author of the letter believes that a visit for one of the minor ailments should be billed as a “partial assessment or subsequent visit”. This definitely was not the view of the physicians with whom we consulted in Saskatchewan. We also consulted with patients who did not have health insurance yet (newcomers to Canada) and hence had to pay out of pocket; they were charged the full amount of $67 by the clinic. We reviewed the Saskatchewan schedule of benefits again and we could not find any clear direction about this and hence it seems that it is up to the physician’s discretion. When we changed this parameter in model from $67 to $35, the net cost saving changed from 3,482,660 to 2,804,861 and ROI changed from 2.53 to 2.04 and hence the program was still cost-saving.

#### Cost of an ER visit

We used the average cost of an ER visit, which we agree may have overestimated the costs of a minor ailment visit to the ER, as these are less serious cases. However, since we assumed that only 3% of the population used ER in the absence of PPMA, varying either cost or wait time for ER has very little/insignificant effect on the results.

#### Wait time for an ER and GP visit

We obtained waiting time for ER based on CIHI report (4.6 h) [13] and we used the value of an individual’s time per hour $ based on statistic Canada ($24.96) [14]. We do not think we should change the value of an individual’s hour based on who is waiting at the ER. We consider this variable as a fixed parameter regardless of socioeconomic level. The author further suggests that we should count the medical examination time as having a different cost than the waiting time to see a doctor, however both times constitute productivity losses for society as the person cannot work during that time.

#### Productivity loss

Contrary to what the author of the letter is claiming, we were quite conservative in our productivity loss estimates, since we only considered only five pharmacist-consulted minor ailments, which accounted for 63% of pharmacist consultations in Saskatchewan. We estimated the number of days absent for each minor ailment based on the literature [15–18] and obtained data from clinical trials and a meta-analysis to estimate the relative effectiveness of each of the most common OTC (OTC drug versus placebo) and prescription medications (prescription versus placebo) for the five minor ailments [18–23].

#### Cost of traveling time

We estimated the average length of time a person spends travelling to the GP clinic and ER based on the geographic distribution of physicians in Canada [24].

#### Five year extrapolation

While we agree that it is difficult to extrapolate the impact of a program over time, we believe it is necessary to provide the current best estimates for the future impact of a project with the data we have and then update the analysis as more information becomes available.

Overall, we were completely transparent about the value of our inputs and our methodology, and therefore if readers are interested in how changes in these parameters impact the results, they can easily change the value of the inputs and see the effect on the results.

### Findings

The author of the letter strongly disagrees with our findings because of a concern that allowing pharmacists to prescribe may lead to overprescribing resulting in both higher costs and lower quality, as indicated in the following three statements in the letter:

- “Whereas a physician is not pressured to provide prescription drugs for every minor condition, a pharmacist must prescribe a drug to get paid.” While, we agree with the author’s concern that business interests must never supersede appropriate clinical decision-making, there is no research evidence suggesting that pharmacists are more likely to prescribe medications than physicians for the same condition. We think it may depend on how physicians are paid as well. Comparative research is needed to investigate this issue.
- *“There may be additional indirect costs in seeking advice within a retail environment, where the patient may be encouraged to purchase additional unproven health products compared with seeking medical attention in a neutral clinic space.”* Pharmacists are proud to be involved in the healthcare of Canadians, of which about 100 years has included the care of minor ailments. Millions of requests for help take place in Canadian pharmacies in any given year. For that involvement, Canadians indicate pharmacists are among the highest of professions in trust. At the University of Saskatchewan, students receive at least 90 h of classroom time exclusively on minor ailments, 16 h of tutorials, and 10 h of practice lab exposure, then practice these skills in summer placements over a four-year program. In each of those contexts, up-selling a patient on something they do not need would severely violate the profession’s ethical standards.
- *“Thus, the PPMA may increase the incidents of adverse drug reactions that physicians would then need to address through additional GP visits.”* This, of course, is possible and must be factored in. As we discuss in our paper, 1.3% of individuals surveyed following a prescribed medication from a pharmacist in Saskatchewan reported side effects [1].

### Policy implications

The author of the letter concludes by saying that these pharmacists prescribing programs may pose a threat to the Canadian health system. We fail to see how allowing pharmacists to prescribe for minor ailments is inconsistent with public health care in Canada or the principles of the Canada Health Act—public administration, comprehensiveness, universality, portability and accessibility [25]. This service is provided free to patients and hence it is publicly and not privately financed; financing is what matters and not provision. This pharmacist prescribing program increases access for patients without imposing any added financial burden on the patient. Since a large proportion of health care providers in Canada work in private clinics [26], this intervention is not a departure from the way the health system is currently organized.

In summary, while we agree with the author that more “evaluations of new services provided by community-based retail pharmacies in Canada” would be very useful, the focus of our study was specifically to perform an economic impact analysis of the pharmacists prescribing for minor ailments program in Saskatchewan. One study and one type of evaluation cannot provide information on all the positive, or negative, consequences associated with a field of study. Our study was very transparent and clear about the research objectives, the analytical approach and input assumptions, and we believe it provides the best estimate to date of the economic impact of a PPMA program in Canada. We look forward to the expanding body of knowledge in this field.

## Abbreviations

PPMA: pharmacists prescribing for minor ailments
EIA: economic impact analysis
OTC: over-the-counter
CMA: cost-minimization analysis
CBA: cost–benefit analysis
ROI: return on investment
