# Correction to: Response to: Costs and savings associated with a pharmacists prescribing for minor ailments program in Saskatchewan

**DOI:** 10.1186/s12962-019-0170-y

**Published:** 2019-01-29

**Authors:** Ellen Rafferty, Mohsen Yaghoubi, Jeff Taylor, Marwa Farag

**Affiliations:** 1 School of Public Health, University of Saskatchewan, Saskatoon, Canada; 2 College of Pharmacy and Nutrition, University of Saskatchewan, Saskatoon, Canada

## Correction to: Cost Eff Resour Alloc (2018) 16:62 10.1186/s12962-018-0159-y

Following publication of the original article [1], these errors were flagged:

1. The article title was missing ‘Response to’.
2. The article was linked to its associated articles incorrectly.

The article [1] has now been corrected accordingly.

Apologies for these errors.
